# Outcome of and Risk Factors for Mortality in Pediatric Coronavirus Disease 2019 Encephalitis During the 2022 Omicron Wave in Taiwan: A National Retrospective Study

**DOI:** 10.1093/ofid/ofag131

**Published:** 2026-03-11

**Authors:** Pei-Jiuan Chao, Wan-Chin Chen, Angela Song-En Huang, Chia-Ping Su

**Affiliations:** Field Epidemiology Training Program, Centers for Disease Control, Ministry of Health and Welfare, Taipei, Taiwan; Division of Infection Control and Biosafety, Centers for Disease Control, Ministry of Health and Welfare, Taipei, Taiwan; Field Epidemiology Training Program, Centers for Disease Control, Ministry of Health and Welfare, Taipei, Taiwan; Department of Infectious Disease, New Taipei Municipal Tucheng Hospital, New Taipei City, Taiwan; School of Medicine, National Tsing Hua University, Hsinchu, Taiwan

**Keywords:** COVID-19, encephalitis, omicron, pediatric, SARS-CoV-2

## Abstract

**Background:**

During Taiwan's severe acute respiratory syndrome coronavirus 2 (SARS-CoV-2) Omicron outbreak in 2022, pediatric coronavirus disease 2019 (COVID-19) encephalitis cases surged, resulting in multiple fatalities. We investigated patient characteristics and mortality risk factors.

**Method:**

Using the Brighton Collaboration criteria, we identified 289 suspected pediatric COVID-19 encephalitis cases diagnosed between April and December 2022 by linking the national surveillance, remdesivir application, immunization, and death registries. We analyzed clinical data and viral genotypes, then used univariate and multivariate logistic regressions to assess mortality risk factors.

**Results:**

We identified 75 encephalitis cases: 50 (67%) were male, with a median age of 5 years (interquartile range [IQR]: 3–8 years), 45 (60%) had no comorbidities, and 23 (31%) had received at least 1 dose of the COVID-19 vaccine. The median interval from symptom onset to medical attention was <1 day (IQR: 0–1 day).

Fourteen cases (19%) died after a median hospital stay of 3 days (IQR: 1–5 days). All sequenced viruses matched the circulating Omicron subvariants. In univariate analysis, age <5 years was associated with increased mortality (odds ratio [OR] & 6.05; 95% confidence interval [CI] & 1.53–24.00). Multivariate analysis showed a nonsignificant trend (adjusted OR & 3.64; 95% CI & 0.81–16.40) after adjustment for sex, comorbidities, and vaccination.

**Conclusions:**

During the Omicron wave, the number of pediatric COVID-19 encephalitis cases progressed rapidly and mortality was high, particularly among children <5 years of age, regardless of comorbidities. We recommend COVID-19 vaccination and monitoring for signs of COVID-19 encephalitis in young children.

## Teaser

We retrospectively examined children <18 years old who had COVID-19 encephalitis during the 2022 Omicron outbreak in Taiwan. We found that the disease progressed quickly and was associated with high mortality, particularly for children <5 years old, regardless of comorbidities.

During 2020–2022, children and adolescents (<20 years) represented 21% of global coronavirus disease 2019 (COVID-19) cases, yet only 0.4% of total deaths, generally exhibiting much lower severity than adults [1, 2]. Despite these broad epidemiological trends, a distinct subset of the pediatric population presented with severe, life-threatening clinical courses that deviate significantly from the typical mild presentation [3–5]. While children with obesity, diabetes, heart disease, chronic lung disease (other than asthma), seizure disorder, or immunocompromised status have a higher risk of severe COVID-19 [6], previously healthy children can experience devastating complications [3–5]. Neurologic involvement is a critical manifestation, reported in 3.8%–44% of hospitalized pediatric patients [7–12]. Although frequently transient, neurologic symptoms result in irreversible deficits or mortality in a significant number of cases [3, 7–9, 11–13].

Owing to the implementation of stringent control measures and nonpharmaceutical interventions from 2020 to 2021, Taiwan had only 1040 COVID-19 patients younger than 18 years (6% of 17 125 total patients) and no deaths within a total population of 2.3 million. However, in December 2021, Taiwan began having imported cases infected with the Omicron variant of severe acute respiratory syndrome coronavirus 2 (SARS-CoV-2) that eventually resulted in widespread community transmission in April 2022 [14–16]. Meanwhile, physicians reported several pediatric deaths due to rapidly deteriorating encephalitis, prompting public health actions. This study investigated the epidemiology and risk factors associated with death among children with COVID-19 encephalitis during the 2022 Omicron variant outbreak in Taiwan, to inform clinical management and public health strategies.

## METHODS

### Study Design and Study Population

The Taiwan Centers for Disease Control (CDC) conducted multifaceted surveillance to detect mild to severe COVID-19 cases and genomic surveillance to track SARS-CoV-2 variants, as described previously [17]. This retrospective case–control study analyzed pediatric COVID-19 patients who had encephalitis with disease onset from April to December 2022. During the study period, COVID-19 was a notifiable infectious disease that had to be reported through the Taiwan National Infectious Disease Reporting System (NIDRS) within 24 hours of diagnosis. We enrolled individuals aged <18 years with confirmed COVID-19 based on positive rapid antigen tests or reverse transcription polymerase chain reaction (RT-PCR) results. To identify pediatric COVID-19 patients with suspected encephalitis, we linked the NIDRS reporting records with remdesivir application forms. Remdesivir is a government-funded antiviral medication that requires formal application. To identify suspected cases, we examined patients whose remdesivir application indicated encephalitis as a condition justifying its use. We also identified deceased patients from the death registry if encephalitis and COVID-19 were recorded as causes of death or underlying diseases that initiated the events leading to death. To ensure data integrity and avoid duplication, we performed deterministic record linkage across the NIDRS, the remdesivir application database, and the national death registry. Unique patient identifiers, including national identification numbers and dates of birth, were used to consolidate disparate records into unique case entries.

### Case Definition for Encephalitis

We reviewed electronic medical records and used the Brighton Collaboration case definition for encephalitis [18]. We classified diagnostic certainty into levels 1 through 5 on the basis of brain histopathology, encephalopathy, focal central nervous system (CNS) abnormalities, and indicators of CNS inflammation (Table 1). We included encephalitis cases classified as having diagnostic certainty levels of 1–3, and we excluded those that lacked medical records or had diagnostic certainty levels of 4 or 5.

**Table 1.** Brighton Collaboration Case Definition of Encephalitis

A. Criterion of Encephalopathy, Focal CNS Abnormalities, Indicators of CNS Inflammation, and Exclusion	
Criterion	Description
Brain histology	Acute inflammation of brain parenchyma
Encephalopathy	Depressed or altered levels of consciousness, lethargy, or personality changes persisting ≥24 h, accompanied by 1 or more of the following criteria: diminished or absent response to stimuli, lack of eye contact, inconsistent or absent reaction to external stimuli, decreased arousability, or seizures coupled with loss of consciousness
Focal CNS abnormalities	Presence of 1 or more of the subsequent features: focal cortical signs, cranial nerve irregularities, visual field defects, primitive reflexes, motor weakness, sensory irregularities, altered deep tendon reflexes, cerebellar dysfunction
Indicators of CNS inflammation	Fever (temperature ≥ 38°C), CSF pleocytosis (>5 WBC/mm^3^ in children aged >2 m; or >15 WBC/mm^3^ in children aged <2 m), EEG results indicative of encephalitis, or neuroimaging results consistent with encephalitis
Exclusion	Alternative diagnoses, such as cancer, vascular disorders, or toxic or metabolic disorders

B. Definitions of Diagnostic Certainty	
Level of Certainty	Description
Level 1	Evidence based on brain histology
Level 2	Presence of encephalopathy or focal CNS abnormalities, combined with 2 or more indicators of CNS inflammation and not meeting the exclusion criteria
Level 3	Presence of encephalopathy or focal CNS abnormalities, combined with 1 indicator of CNS inflammation and not meeting the exclusion criteria
Level 4	Reported as encephalitis but insufficient evidence to meet any level of case definition and not meeting the exclusion criteria
Level 5	Meeting the exclusion criteria or lacking any evidence, including brain histopathology, encephalopathy, and focal CNS abnormalities

Note: Case definitions were adapted from the Brighton Collaboration encephalitis criteria [18]. Abbreviations: CNS, central nervous system; CSF, cerebrospinal fluid; EEG, electroencephalography; WBC, white blood cells.

### Data Collection

Information we collected from the NIDRS and electronic medical records included age and sex; dates of disease onset, hospital admission discharge, and death; underlying conditions; history of COVID-19; clinical symptoms; laboratory and neurologic examination results; treatment courses; and discharge status. Due to data incompleteness in a subset of the patients, the analysis of therapeutic interventions was restricted to patients with comprehensive medical records. Causes of death and clinical complications were ascertained from discharge diagnoses and official physician-certified death certificates. We defined “underlying conditions’ as any pre-existing diagnosis documented in the medical record, regardless of chronicity. While a single, uncomplicated febrile convulsion is generally not classified as a chronic neurologic disorder, it was included in our analysis to ensure comprehensive clinical capture. We retrieved virus variant types from the SARS-CoV-2 genomic surveillance system and retrieved history of COVID-19 vaccination from the national immunization registry, which contained all COVID-19 vaccination records. Coronavirus disease 2019 vaccine eligibility was determined by age before the onset of COVID-19 diagnosis, because Taiwan had an age de-escalation schedule for COVID-19 vaccination and not all children were eligible to receive the COVID-19 vaccine. Height and weight were extracted from admission records, and weight percentiles were determined from the growth curves for children, published by the Taiwan Pediatric Association [19].

### Statistical Analysis

Continuous variables are expressed as median and interquartile range (IQR). Categorical variables are summarized as frequencies and percentages. Univariate regression analyses were performed to determine risk factors associated with mortality, with results presented as odds ratios (ORs), 95% confidence intervals (CIs), and *P* values. Multivariate regression analysis incorporated risk factor *P* values of <.2 from the univariate regression analyses. Data analyses were conducted with Epi Info software, version 7.2.6.0. Variables with a 2-sided *P* value of <.05 were considered statistically significant.

### Ethical Approval

This study was approved by the Institutional Review Board of the Taiwan CDC, Ministry of Health and Welfare, Taiwan (approval number: 112205), and a waiver of informed consent was granted, as the study involved minimal risk and was conducted as part of fulfilling statutory duties of the government agency, with appropriate safeguards in place to protect data confidentiality. All patient data included in the study were fully anonymized.

## RESULTS

### Study Population

**Figure 1.**
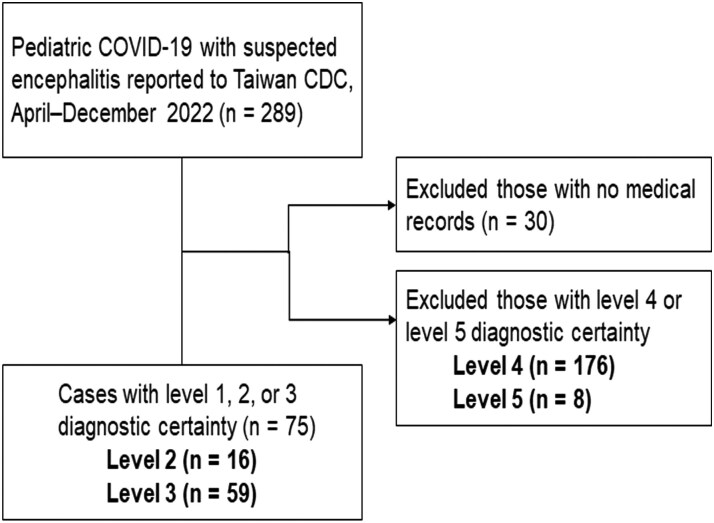
Case selection flow chart for pediatric COVID-19 encephalitis.

Between April and December 2022, 1 675 069 confirmed pediatric COVID-19 cases were reported to the Taiwan CDC, including 49 pediatric deaths. We identified 289 suspected pediatric cases of COVID-19 encephalitis. After excluding 30 that lacked medical records and 184 with a diagnostic certainty level of 4 or 5, we enrolled 75 encephalitis cases, comprising 16 cases at level 2 and 59 at level 3 (Figure 1).

**Figure 2.**
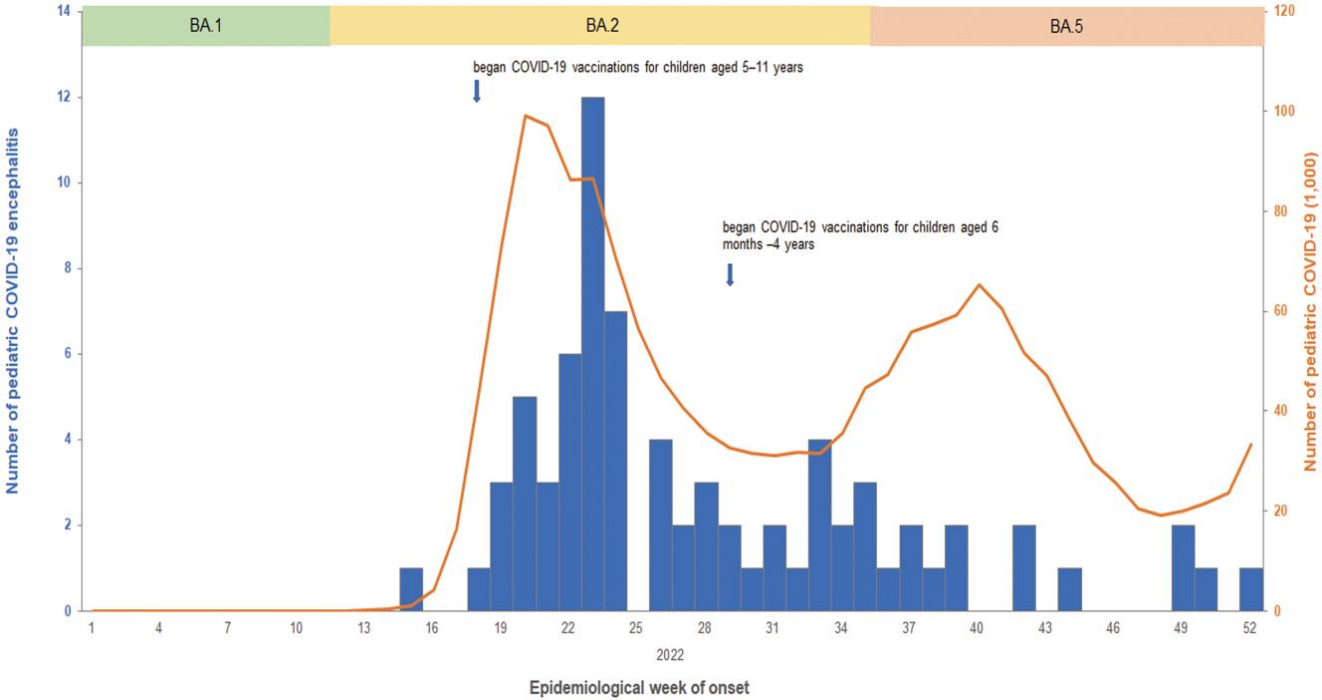
Weekly pediatric COVID-19 and encephalitis cases in Taiwan, 2022 (n = 75). The bar chart indicates the number of pediatric COVID-19 encephalitis cases, while the line plot represents the number of pediatric COVID-19 cases (normalized per 1000 children); both are plotted according to the epidemiological week of onset. During this period, the predominant circulating virus shifted, from Omicron variant BA.1 subvariant in weeks 1–11 to BA.2 in weeks 12–34 and BA.5 in weeks 35–52. Pediatric encephalitis cases increased along with an increase in COVID-19 cases during the BA.2 wave. Following vaccination of children aged 5–11y and later those 6 m–4 y, no increase in encephalitis cases was observed when COVID-19 cases increased during the BA.5 wave. Note: COVID-19 vaccination for adolescents aged 12–17 y began in September 2021 (not shown in this figure).

Pediatric encephalitis cases increased during epidemiological week (Epi Week) 19–23, coinciding with the peak of pediatric COVID-19 caused by the BA.2 subvariant of Omicron (Figure 2), with 55% (41/75) occurring in May and June. However, when the BA.5 subvariant became dominant after Epi Week 35, encephalitis cases did not surge, despite an increase in COVID-19 cases.

By Epi Week 13, COVID-19 vaccination was available only to those aged 12 years and above, and 81% of adolescents aged 12–17 years received 2 doses.

Vaccination eligibility was expanded to include children 5–11 years of age in Epi Week 18 and 6 months–4 years of age in Epi Week 29. By the end of 2022, 2 dose vaccination rates reached 88% among those aged 12–17 years, 65% among those aged 5–11 years, and 23% among those aged 6 months–4 years.

### Demographics and Clinical Characteristics of Encephalitis Cases

Of the 75 patients with encephalitis, 50 (67%) were male; the median age was 5 years (IQR: 3–8 years), and 45 (60%) had no underlying disease. Only 1 patient had a previous COVID-19, with a 4-month interval between infections. Fifty-two (69%) of the patients were unvaccinated, of which 30 (58%) were vaccine eligible. The most common symptoms were fever (n & 74, 99%), altered consciousness (n & 59, 79%), and seizures (n & 51, 69%). Neuroimaging showed signs of encephalitis in 14 of 65 cases (22%), and electroencephalography (EEG) findings were consistent with encephalitis in 3 of 21 cases (16%). Among the 19 cases in which the cerebrospinal fluid was analyzed, 3 (16%) had pleocytosis. Viral genome sequencing was available for 18 cases and revealed subvariants of Omicron (16 BA.2 and 2 BA.5), which were genetically identical to the circulating community variants during the corresponding period (Figure 2). Forty-three cases (57%) required intensive care, and the most common anti-SARS-CoV-2 therapy was administration of remdesivir (n = 72, 97%) followed by steroids (54/66, 82%). The median interval between symptom onset and the seeking of medical attention was less than 1 day (IQR: 0–1 day). The median hospital stay was 5 days (IQR: 4–7 days) (Table 2). Upon discharge, 14 patients (19%) died, and 2 of the survivors had limb weakness (1 case with quadriplegia and 1 case with hemiplegia).

**Table 2.** Characteristics of Pediatric COVID-19 Encephalitis Cases and Risk Factors for Mortality

Variable	Total	Died	Survived	Univariate		Multivariate	
	(n = 75) (%)	(n = 14) (%)	(n = 61) (%)	OR (95% CI)	*P*	OR (95% CI)	*P*
Sex							
Female	25 (33)	7 (50)	18 (30)	2.39 (0.73–7.81)	0.15	1.87 (0.51–6.95)	0.34
Age							
Median (IQR), y	5 (3–8)	3 (2–8)	5 (3–8)	0.84 (0.69–1.02)	0.07	—	—
<5 y	24 (32)	11 (79)	23(38)	6.05 (1.53–24.00)	0.01	3.50(0.79–15.57)	0.10
Body weight percentile							
<3rd	4/68 (6)	1 (7)	3/54 (6)	—	—	—	—
>97th	10/68 (15)	1 (7)	9/54 (17)	—	—	—	—
3rd–97th	54/68 (79)	12 (86)	42/54 (78)	1.71(0.34–8.72)	0.52	—	—
Underlying condition(s)^a^							
No	45 (60)	11 (79)	34 (56)	2.91(0.74–11.49)	0.13	2.17(0.49–9.59)	0.31
Yes	30 (40)	3 (21)	27 (44)	—	—	—	—
Febrile convulsion	11 (15)	1 (7)	10 (16)	—	—	—	—
Developmental delay	8 (11)	1 (7)	7 (11)	—	—	—	—
Asthma	6 (8)	1 (7)	5 (8)	—	—	—	—
Psychiatric disease	3 (4)	0 (0)	3 (5)	—	—	—	—
Congenital anomaly	2 (3)	0 (0)	2 (3)	—	—	—	—
Epilepsy	2 (3)	0 (0)	2 (3)	—	—	—	—
Allergic rhinitis	2 (3)	0 (0)	2 (3)	—	—	—	—
Thalassemia	2 (3)	0 (0)	2 (3)	—	—	—	—
Cerebral palsy	1 (1)	0 (0)	1 (2)	—	—	—	—
Viral hepatitis B carrier	1 (1)	0 (0)	1 (2)	—	—	—	—
Atopic dermatitis	1 (1)	0 (0)	1 (2)	—	—	—	—
COVID-19 vaccination status							
Unvaccinated	52 (69)	13 (93)	39 (64)	7.33(0.90–59.88)	0.06	2.98(0.31–28.97)	0.35
Vaccinated (1 or 2 doses)	23 (31)	1 (7)	22 (36)	—	—	—	—
Symptom on presentation							
Fever	74 (99)	14 (100)	60 (98)	—	—	—	—
Change of consciousness	59 (79)	14 (100)	45 (74)	—	—	—	—
Seizure	51 (69)	12 (86)	39 (64)	—	—	—	—
Hallucination	25 (33)	2 (14)	23 (38)	—	—	—	—
Ataxia	5 (7)	1 (7)	4 (7)	—	—	—	—
Aphasia	3 (4)	0 (0)	3 (5)	—	—	—	—
Investigation (findings suggestive of)							
Neuroimaging (encephalitis)	14/63 (22)	5/13 (38)	9/50 (18)	—	—	—	—
CT	5/60 (8)	4/13 (31)	1/47 (2)	—	—	—	—
MRI	10/18 (56)	2/2 (100)	8/16 (50)	—	—	—	—
EEG (encephalitis)	3/21 (14)	1/2 (50)	2/19 (11)	—	—	—	—
CSF (pleocytosis)	3/19 (16)	1/4 (25)	2/15 (13)	—	—	—	—
SARS-CoV-2 genomic sequencing done							
No	57 (76)	10 (71)	47 (77)	—	—	—	—
Yes	18 (24)	4 (29)	14 (23)	—	—	—	—
BA.2	16 (21)	3 (21)	13 (21)	—	—	—	—
BA.5	2 (2)	1 (7)	1 (2)	—	—	—	—
Medical treatment status, median days; IQR							
Between onset and seeking treatment	0; 0–1	0; 0–1	0; 0–1	**—**	—	—	—
Between onset and admission	1; 0–2	1; 1–2	1; 0–2	—	—	—	—
Hospital stay	5; 4–7	3; 1–5	6; 5–7	—	—	—	—
Treatment							
Remdesivir	72 (97)	12 (86)	60 (98)	—	—	—	—
Intravenous immunoglobulin	39/63 (62)	9/13 (69)	30/50 (60)	—	—	—	—
Steroids	54/66 (82)	13/13 (100)	41/53 (77)	—	—	—	—
Tocilizumab	22/63 (35)	9/13 (69)	13/50 (26)	—	—	—	—

Abbreviations: CI, confidence interval; CSF, cerebrospinal fluid; CT, computed tomography; EEG, electroencephalography; IQR, interquartile range; MRI, magnetic resonance imaging; OR, odds ratio.

^a^Patients may have had more than 1 underlying condition.

### Demographics and Clinical Characteristics of Fatal Cases

Of the 14 patients who died, 7 were male (50%), with a median age of 3 years (IQR: 2–8 years); only 3 (21%) had underlying diseases. The median hospital stay was 3 days (IQR: 1–5 days), and the median duration from symptom onset to death was 5 days (IQR: 4–6 days). None of the cases had out-of-hospital cardiac arrest, 13 (93%) required intensive care, and 4 died within 1 day of hospitalization (Table 2). Neurological complications included brain edema (n = 7, 50%), central diabetes insipidus (n = 5, 36%), acute necrotizing encephalopathy during childhood (n = 2, 14%), acute disseminated encephalomyelitis (n = 1, 7%), and neurogenic shock (n = 1, 7%). Other complications included multiple organ failure (n = 5, 36%), pneumonia (n = 4, 29%), gastrointestinal hemorrhage (n = 3, 21%), diffuse intravascular coagulation (n = 3, 21%), myocarditis (n = 1, 7%), and ventricular tachycardia (n = 1, 7%) (Supplementary Table 1). Among the fatalities, only 1 individual had received prior COVID-19 vaccination; this 8-year-old male with no underlying diseases became ill 6 months after receiving 2 doses of COVID-19 vaccine and died 4 days after symptom onset.

### Risk Factors Associated With Mortality

In the univariate regression analysis, age <5 years (OR, 6.05; 95% CI, 1.53–24.00; *P* & .01) was associated with mortality (Table 2). Among the 51 vaccine-eligible children, 28 (54.9%) remained unvaccinated. The mortality rate was 14.3% (4/28) in the unvaccinated group, compared to 4.3% (1/23) among those vaccinated. Univariate analysis revealed that children who were eligible but unvaccinated had a higher risk of mortality (OR, 3.67; 95% CI, 0.38–35.36; *P* = .36), but the association did not reach statistical significance. Multivariate regression analysis, which was adjusted for sex, underlying conditions, and COVID-19 vaccination status, indicated that age <5 years had a trend toward association with mortality, but this did not reach statistical significance (adjusted OR, 3.50; 95% CI, 0.79–15.57; *P* = .10) (Table 2).

## DISCUSSION

During the 2022 Omicron outbreak in Taiwan, among pediatric patients with COVID19 complicated by encephalitis, the fatality rate was high (19%), and most of those who died were <5 years of age. Most patients were previously healthy and did not receive COVID-19 vaccination. Despite prompt antiviral treatment, their conditions rapidly deteriorated. Most deaths occurred in the context of fulminant encephalitis, frequently accompanied by multiorgan failure and shock. A small proportion of survivors had persistent neurologic deficits upon discharge.

Neurologic complications among children hospitalized with COVID-19 are associated with an increased risk of intensive care unit admission and in-hospital mortality [11, 20, 21]. Various studies have shown variability in the incidence and outcome of neurologic involvement among hospitalized children with acute COVID-19 during the pre-Omicron period. In the United States, multicenter studies showed that 22%–28% of children and adolescents hospitalized for acute COVID-19 exhibited neurologic involvement; of these neurologic findings, approximately 10% were life threatening, including encephalitis. The case fatality rate was up to 35% among those with severe neurologic complications, and 36%–43% of survivors had new neurologic deficits at discharge. Despite being eligible for vaccination, most patients with severe COVID-19-associated neurologic involvement were unvaccinated [8]. A multinational prospective cohort study similarly found severe neurologic manifestations in 18% of hospitalized children with acute COVID-19, which were significantly associated with new cognitive and functional impairments at discharge; 5% of these patients died in the hospital [22].

Contrastingly, a national prospective study in the United Kingdom during the first 9 months of the COVID-19 pandemic revealed no fatalities among pediatric patients with acute COVID-19 and neurologic manifestations, although 37% of these patients had neurologic impairments upon discharge [9]. These variations likely reflect differences in patient populations, healthcare resources during the pandemic, definitions of neurologic complications, and methodologic approaches, highlighting the importance of focused studies like ours that specifically investigated pediatric encephalitis related to acute COVID-19.

During the Omicron variant transmission era, characterized by increased transmissibility but reduced virulence, Japan and Korea reported significant pediatric mortality associated with neurologic complications despite expanded eligibility for COVID-19 vaccination among children. In Japan, from January to September 2022, CNS abnormalities were attributed to 38% (19/50) of pediatric COVID-19-related deaths. Among those patients who died, 47% (9/19) were male, with a median age of 8 years (IQR: 3–10 years); 58% of the pediatric COVID-19-related deaths involved no underlying medical conditions, and only 6% of the deaths were of patients who received a vaccine. Japan also reported rapid disease progression from symptom onset to death, with a median duration of 2 days (IQR: 1–12 days), despite appropriate therapies [3]. Similarly, the Korea Disease Control and Prevention Agency reported an increase in pediatric COVID-19 deaths in 2022. As of 3 September 2022, among the 22 (of 46 pediatric deaths) with documented causes of death, 2 were due to severe brain edema and 2 to encephalitis [4]. Another multicenter Korean study, conducted from January to April 2022, identified 8 pediatric patients with severe neurologic manifestations associated with acute COVID-19. Among them, 38% (3/8) were male, and the median age was 9 years (IQR: 4–18 years). Significant morbidity and mortality were observed among unvaccinated children and those with pre-existing neurologic disabilities [13]. In contrast, our study found that the median age of fatal cases was lower than that reported in Japan and Korea. While previous studies have suggested that older children may be at a greater risk for neurologic complications [11, 20], the younger age observed in our study may be partly due to the lower vaccination rates among children <5 years old. Additionally, young children have a more permeable blood–brain barrier, which may facilitate the entry of inflammatory cytokines and viruses into the brain, potentially contributing to neurologic damage [23].

The pathogenesis of COVID-19 that leads to neurologic complications is not yet fully understood. Although the short study period might limit the findings, the absence of an association between the Omicron variant and neurologic complications in studies in the United States suggests potential genetic susceptibility differences in the virus across populations [20]. Notably, acute necrotizing encephalopathy following viral infections has been more frequently reported in Asian children than in Western countries [24]. Genetic susceptibility to acute encephalopathy and encephalitis in East Asian populations, especially in Japan and China, has been linked to thermolabile mutations in the carnitine palmitoyltransferase II gene [25, 26]. Furthermore, a recent study implicated neutrophil-mediated inflammatory responses as contributing factors to cerebral dysfunction in Omicron BA.2-related encephalitis [27]. Future studies on the genetic, immunological, and inflammatory pathways specific to pediatric COVID-19 encephalitis are urgently needed.

Encephalitis is a rare but life-threatening neurologic complication in pediatric COVID-19 patients [28, 29]. While studies have identified infants and the presence of underlying health conditions as key factors influencing disease severity in children [6, 30–32], our cohort showed that 60% of the cases involved previously healthy patients, and only 31% had received at least 1 dose of COVID-19 vaccination.

Before the SARS-CoV-2 Omicron variant outbreak in Taiwan began in April 2022, COVID-19 vaccines were available only for individuals aged ≥12 years. Even after emergency authorization for younger age groups, two-dose coverage among children aged 6 months to 4 years remained low until the end of 2022. We found that fatal encephalitis was more common in unvaccinated children <5 years of age. Large-scale studies have demonstrated that the COVID-19 vaccine substantially reduces hospitalizations and deaths in pediatric populations [33, 34]; however, our regression analysis did not identify a statistical significance for vaccination status, most likely reflecting both low vaccine uptake and an insufficient number of vaccinated versus unvaccinated patients. Throughout the COVID-19 pandemic, healthy children are usually considered a low priority for vaccination because they are at lower risk for mortality following infection and constrained vaccine supply [35–37]. Nevertheless, given the high mortality associated with COVID-19 encephalitis and the predominance of cases involving healthy, unvaccinated children, promoting COVID-19 vaccination in all children remains critical in reducing the risk of infection and death from encephalitis, for which effective treatments are currently limited.

Our study had several limitations. First, our sampling strategy, which relied on remdesivir applications and death registries, likely introduces a selection bias toward severe cases. Consequently, children with milder COVID-19 encephalitis—those failing to meet remdesivir application criteria or managed via alternative protocols—might have been underrepresented. This potential selection bias may lead to an overestimation of the case fatality rate among all children with COVID-19-associated encephalitis. Second, an underestimation of encephalitis may have occurred because of reliance on comprehensive medical records for Brighton Collaboration diagnostic certainty levels, potentially resulting in a selection bias. Third, given the retrospective nature of this study and the fulminant clinical course of the affected children, it was difficult to differentiate whether systemic complications—specifically multiorgan failure and shock—were direct sequelae of severe neurologic insult or independent contributing factors. These pathologies frequently presented concomitantly, precipitating rapid clinical decompensation. Fourth, the limited number of cases may constrain the statistical power and affect the generalizability of the findings. Finally, age and vaccination status were closely correlated, complicating the interpretation of independent effects.

In conclusion, pediatric COVID-19 encephalitis during the Omicron wave in Taiwan was characterized by rapid disease progression and a high case fatality rate. We recommend the promotion of COVID-19 vaccination for all children and increasing clinician awareness of encephalitis symptoms in children with COVID-19, particularly among patients <5 years old.

## Supplementary Material

ofag131_Supplementary_Data
