# Supplementary material for: Outcome of and Risk Factors for Mortality in Pediatric Coronavirus Disease 2019 Encephalitis During the 2022 Omicron Wave in Taiwan: A National Retrospective Study
Source: Open Forum Infect Dis. 2026 Mar 11;13(3):ofag131. doi: 10.1093/ofid/ofag131 (PMC13014466; doi:10.1093/ofid/ofag131)
Supplement: ofag131_Supplementary_Data [file ofag131_supplementary_data.docx]

**Supplementary Table 1**. Clinical, diagnostic, and features of fatal cases of pediatric COVID-19 encephalitis, 2022 (n = 14)

| **Case No.** | **Sex** | **Age (years)** | **Underlying conditions** | **COVID-19 vaccine (dose)** | **Clinical Presentation** | **Days from onset to death** | **Brighton diagnostic certainty** | **Neuroimage findings (CT/MRI)** | **EEG** | **Neurologic complications** | **Other complications** |
| --- | --- | --- | --- | --- | --- | --- | --- | --- | --- | --- | --- |
| #1 | M | 2 | No | 0 | Fever, altered consciousness, seizure, dyspnea | 6 | 2 | Brain edema | Not performed | Brainstem encephalitis and acute diffuse cerebral edema | DIC |
| #2 | F | 4 | No | 0 | Fever, altered consciousness, convulsion, rhinorrhea, cough, visual hallucination | 11 | 3 | Brain edema | Not performed | Brain edema | Septic shock with multiple organ dysfunction, acute fulminant hepatitis |
| #3 | M | 2 | No | 0 | Fever, altered consciousness, convulsion | 2 | 3 | Not performed | Not performed | ANEC | Multiple organ dysfunction |
| #4 | M | 10 | No | 0 | Fever, altered consciousness, seizure, vomiting, cough, visual hallucination | 5 | 2 | Brain edema | Not performed | Brain edema | Multiple organ dysfunction, ventricular tachycardia, pneumonia |
| #5 | M | 3 | No | 0 | Fever, altered consciousness, general weakness | 4 | 2 | ANEC, with brainstem, thalami involvement | Not performed | ANEC with neurogenic shock and CDI | Acute hepatitis, gastrointestinal hemorrhage |
| #6 | F | 9 | Asthma | 0 | Fever, altered consciousness, ataxia | 88 | 2 | Brain edema, ADEM | Electroclinical seizures and focal epileptiform discharge | Hemorrhagic ADEM, brainstem injury | Urinary tract infection |
| #7 | F | 4 | Febrile convulsion | 0 | Fever, altered consciousness, seizure, lethargy | 5 | 3 | Brain edema | Not performed | CDI | Acute hepatitis, acute kidney injury, gastrointestinal hemorrhage |
| #8 | F | 1 | No | 0 | Fever, altered consciousness, seizure | 1 | 3 | Suspicious faint lower density changes in midbrain | Not performed | Brainstem encephalitis | Pneumonia, shock |
| #9 | M | 3 | Developmental delay | 0 | Fever, altered consciousness, dyspnea, diarrhea | 5 | 2 | Low density changes of bilateral thalami | Profound cerebral dysfunction | Fulminant encephalitis with cerebral edema and CDI | Acute myocarditis, acute hepatitis, acute kidney injury, DIC, sepsis |
| #10 | F | 4 | No | 0 | Fever, altered consciousness, irritability | 4 | 3 | Suspect small size of the bilateral lateral ventricles | Not performed | _ | Pneumonia, hyperammonemic encephalopathy |
| #11 | F | 3 | No | 0 | Fever, altered consciousness, cough, diarrhea | 2 | 2 | Suspect encephalitis in left medial temporal lobe | Not performed | _ | Shock |
| #12 | F | 2 | No | 0 | Fever, altered consciousness, cough, rhinorrhea, vomiting | 4 | 2 | ANEC | Not performed | ANEC, severe dysautonomia and uncal herniation | Multiple organ dysfunction, shock |
| #13 | M | 1 | No | 0 | Fever, altered consciousness, seizure | 3 | 3 | Brain edema | Not performed | CDI, brain edema | Shock, upper gastrointestinal hemorrhage, pneumonia |
| #14 | M | 8 | No | 2 | Fever, altered consciousness, seizure, abdominal pain, vomiting | 4 | 3 | Brain edema | Not performed | CDI, brain edema | Pneumonia, acute pulmonary edema, shock, acute kidney injury, DIC |

ADM, acute disseminated encephalomyelitis; ANEC, acute necrotizing encephalopathy of childhood; CDI, central diabetes insipidus; CT, computed tomography; DIC, disseminated intravascular coagulation; EEG, electroencephalography; MRI, magnetic resonance imaging.
